# An automated rat grimace scale for the assessment of pain

**DOI:** 10.1038/s41598-023-46123-x

**Published:** 2023-11-01

**Authors:** Brendan Arnold, Rahul Ramakrishnan, Amirah Wright, Kelsey Wilson, Pamela J. VandeVord

**Affiliations:** 1 School of Biomedical Engineering and Sciences, Virginia Tech, Blacksburg, VA USA; 2 Academy of Data Science, Virginia Tech, Blacksburg, VA USA; 3 Veterans Affairs Medical Center, Salem, VA USA; 4 Department of Biomedical Engineering and Mechanics, Virginia Tech, 440 Kelly Hall, 325 Stanger St., Blacksburg, VA 24060 USA

**Keywords:** Biomedical engineering, Brain injuries, Headache, Neuropathic pain

## Abstract

Pain is a complex neuro-psychosocial experience that is internal and private, making it difficult to assess in both humans and animals. In pain research, animal models are prominently used, with rats among the most commonly studied. The rat grimace scale (RGS) measures four facial action units to quantify the pain behaviors of rats. However, manual recording of RGS scores is a time-consuming process that requires training. While computer vision models have been developed and utilized for various grimace scales, there are currently no models for RGS. To address this gap, this study worked to develop an automated RGS system which can detect facial action units in rat images and predict RGS scores. The automated system achieved an action unit detection precision and recall of 97%. Furthermore, the action unit RGS classifiers achieved a weighted accuracy of 81–93%. The system’s performance was evaluated using a blast traumatic brain injury study, where it was compared to trained human graders. The results showed an intraclass correlation coefficient of 0.82 for the total RGS score, indicating that the system was comparable to human graders. The automated tool could enhance pain research by providing a standardized and efficient method for the assessment of RGS.

## Introduction

Pain is a complex multifaceted experience influenced by a range of biological, psychological, and social factors. Furthermore, it is a subjective experience that can be challenging to quantify and measure objectively in both humans and animals^1,2^. Despite the difficulties associated with assessing pain, it is a crucial aspect of research in elucidating the mechanisms of injuries and their potential treatments. This research can encompass studies on pain mechanisms such as, the role of neurotransmitters, nerve pathways, and other biological processes, as well as research on potential instruments to alleviate the underlying processes. However, conducting research on human subjects is very challenging. This is partly due to ethical considerations and limited ability to congregate a group of subjects with similar conditions without pronounced confounding variables. Nevertheless, advancing our understanding of pain and developing new treatments would provide a better quality of life for millions worldwide. Therefore, animal research is paramount in investigating pain’s mechanisms for improved treatment.

In preclinical research approximately 95% of animal models use rodents, with rats being among the most common for pain studies^3^. Rat models are used in pain research due to the many similarities they share with humans in their physiology and pain pathways^4^. These similarities enable researchers to study injury response and treatment in rats and to relate the finding to human response, prior to clinical studies. Despite the advantages of rodents in research, they are still non-verbal beings that cannot directly communicate their experiences. To compensate for this limitation, researchers use a range of tests to assess the effectiveness of a treatment or the extent of an injury. These include assessments of response to nociceptive stimuli such as mechanical, thermal, and chemical responses as well as behavioral tests including the tracking of movement, weight loss, and socialization^5^. While these tests can provide useful information on the wellbeing of the rat, traditional assessments of the pain response struggle to demonstrate a direct connection between behavior and pain experience. In many of these tests, the secondary response to pain is monitored such as latency for withdrawal from a stimulus, rather than the pain experience itself.

One solution for this problem is the use of facial action coding systems (FACS). FACS are coding systems which analyze facial movements relevant to emotion. By assessing specific facial movements under known emotional contexts researchers can help identify particular action units for emotional expression and use them as a systematic measurement to strongly suggest emotional states without relying on verbal communication. FACS have demonstrated utility among nonverbal humans such as infants and those with communication disabilities^6–8^. Building on the success in nonverbal humans, further coding systems were developed for a variety of animals to provide a direct measurement of pain.

Langford et al. conducted one of the earliest studies to evaluate pain response in non-human mammals, using laboratory mice^9^. This study performed a preclinical pain assay of 0.9% acetic acid abdominal constriction test to determine pain related action units and develop a mouse grimace scale (MGS). After introducing the pain stimulus, the mice were filmed for 30 min, and facial images were captured at 3-min increment. Using these images, they created a FACS to measure pain expression through orbital tightening, nose bulging, cheek bulging, ear positioning, and whisker changes. A secondary study was then conducted where the MGS was applied to a mouse migraine model which found an elevated MGS score in the mutant mice compared to wild-type mice. The success of the MGS led to the development of grimace scales for other species, including felines^10^, horses^11^, and pigs^12^. However, one of the most significant developments was the rat grimace scale (RGS), which provided researchers with a primary measure of pain in one of the most prevalent pain models in research^3,13^.

RGS was developed shortly after MGS in a study by Sotocinal et al. to aid in the quantification of the rat’s pain response using facial expressions^13^. RGS uses four action units including orbital tightening, nose flattening, ear changes, and whisker changes and scores them on a 0–2 scale to numerically evaluate the rat’s wellbeing. During a RGS experiment, the rat is placed in a clear chamber, and they are recorded with a frontal-facing camera. The frontal images of the rat are then taken from specified timepoints throughout the video and individually graded by a team of trained researchers.

According to Mogil et al. the usage of the RGS and the MGS has been on the rise since their development^14^. Initially designed for acute pain, RGS has been applied to chronic pain studies, where it has shown elevated scores long after the initial stimulus^15–18^. Despite its effectiveness, using RGS can be challenging due to the time-consuming image collection, grading, and training involved. Additionally, research has found that inter-rater reliability of RGS, measured by the intraclass correlation coefficient (ICC), is high for orbital tightening but less so for other action units^16,19,20^. Increasing the number of human graders might address this issue, but the required training and time commitment makes this difficult to implement. Moreover, many studies using RGS do not provide details about the rater’s training or inter-rater agreement, undermining the reliability of their results^14^. For these reasons research into machine learning and computer vision techniques for grimace scale grading is currently being explored.

Various machine learning approaches have been used to automate the grimace scale for several animals including sheep^21^, horses^22^ and felines^23^. However, the largest focus has been on the MGS, which has an automated program (aMGS) using a deep convolutional neural network (CNN)^24^. Furthermore, additional research has taken place to improve upon the aMGS’s accuracy^24,25^. Despite the resources being implemented into the improvement of the MGS system, no fully automated system has been developed for RGS regardless of the widespread use of rats in pain studies.

This study aimed to address this gap by developing an automated RGS (aRGS) scoring system based on object detection and classification models commonly used in computer vision. The first objective of this study was to create an aRGS scoring system that can detect facial action units within a frontal-facing image of a rat subjected to repeated blast-induced traumatic brain injury (bTBI) and grade them according to the RGS standards. Traumatic brain Injury (TBI) in humans, including from blast exposure, is associated with increased sensitivity to painful stimuli and chronic pain^26–28^. Preclinical models have shown pain related in rats subjected to TBI with spontaneous pain analysis completed with RGS^17,18^. Due to the difficulty of preclinical model pain assessments, an aRGS would be a valuable tool for researchers. The second aim was to then validate this program against manually graded RGS images from trained scorers for accuracy and intergrader reliability. The development of an automated system for RGS scoring would greatly enhance the efficiency of RGS as well as reduce the barriers to entry which may prevent studies from utilizing the RGS system.

## Results

### Dataset

The database included a total of 1122 frontal-facing images of adult (aged 8 weeks or older) male and female Sprague Dawley and Wistar rats. These images were used to further develop secondary action unit databases containing 1482 eye images, 1363 ear images, and 1117 nose images. The frontal images were sourced from various RGS setups to ensure the prediction model could be generalized for different RGS imaging protocols. The majority of the database included images from RGS testing including Furman et al.^29^ where rats were induced with neuropathic orofacial pain by chronic constriction injury of the infraorbital nerve, as well as Zhang et al.^20^ which developed a RGS dataset to evaluate interrater reliability. Additionally, the database was supplemented with frontal-facing rat images taken during periorbital Von Frey testing to increase the samples of elevated RGS action units.

The images were then annotated for object detection where bounding boxes were drawn around clearly visible ears and eyes that could be viably used for RGS scoring. For the nose, a bounding box was drawn from the upper cheek bone below the eyes to the tip of the nose, as long as it was not obstructed. Moreover, each action unit image was annotated with their respective RGS scores for orbital tightening, ear changes, and nose/cheek flattening.

In addition to the studies listed above, unpublished data collected by our laboratory independently validated the induction of pain-like behaviors post blast by manual von-frey testing (IACUC Protocol ID 21-218). Thus, additional datasets were established using a validated injury model for RGS testing of blast traumatic brain injury (bTBI)^17,18^. This validation study included a control group of naive 10-week-old male Sprague Dawley rats (n = 10) and a group subjected to bTBI (n = 10), followed by RGS imaging described in Sotocinal et al.^13^. The validation set was comprised of 40 control images of naïve rats, 40 images from the existing bTBI study at day one of injury, and 40 images of 1-month post-injury taken with a GoPro HERO8 Black.

### Action unit detection

**Figure 1:**
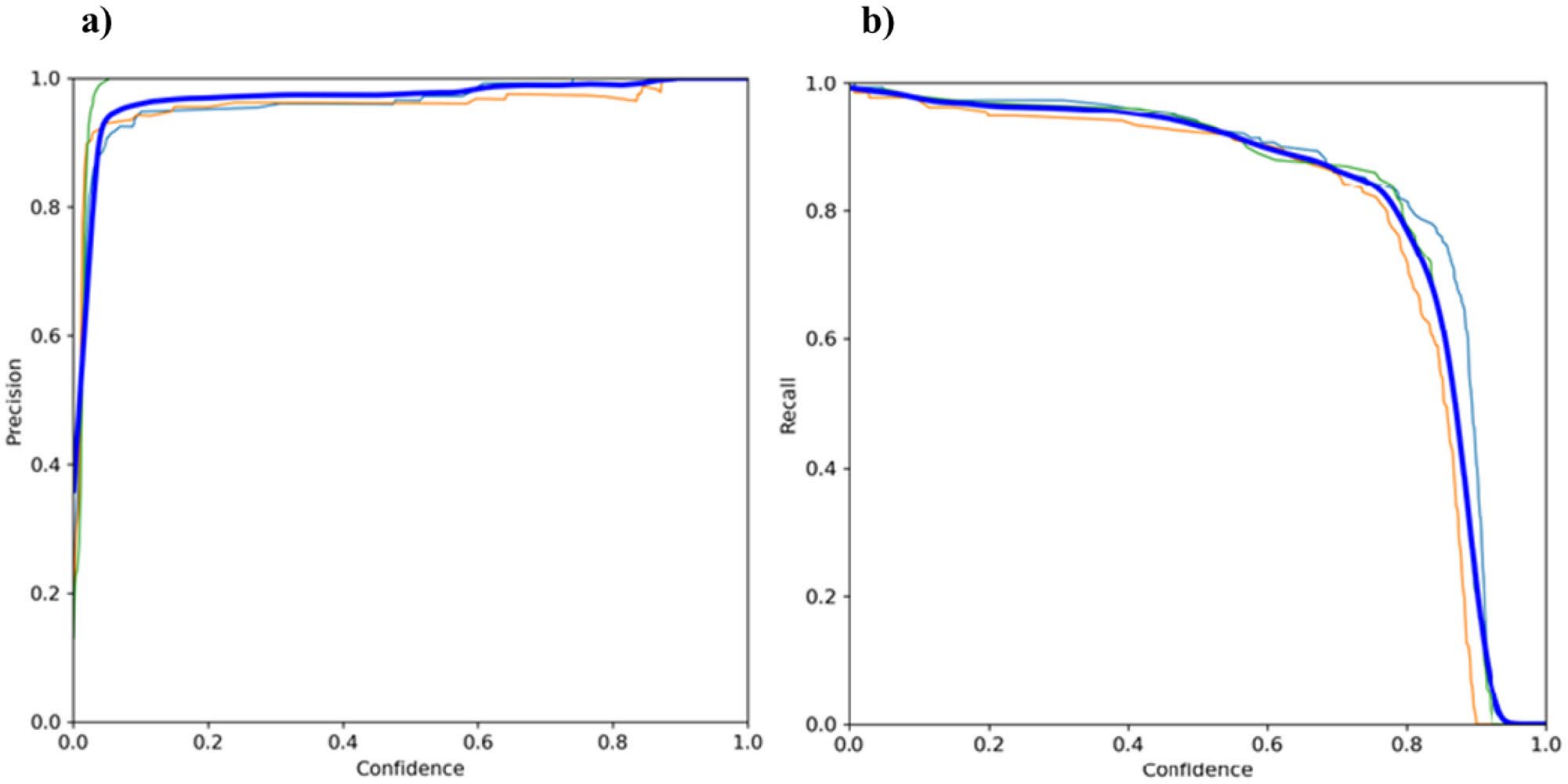
YOLOv5 final model metrics for global features (bolded blue), eyes (orange), ears (light blue), nose (green): (**a**) Precision-Confidence Curve; (**b**) Recall-Confidence Curve.

A YOLOv5 model was fine-tuned for the detection of facial action unit areas such as the eyes, ears, and nose. Taking an image as an input, the YOLO algorithm used a simple CNN to detect objects in the image using the Leaky ReLU activation function for the hidden layers and the sigmoid activation function for the final detection layer^30^. Additionally, the model used stochastic gradient descent (SGD) with momentum for the optimization function^30^. Three loss functions (box regression, objectness loss, and class loss) were calculated with the weighted sum between the three losses used as the overall loss function^30^. Box loss was weighted as 0.05, object loss was weighted as 1, and class loss was weighted as 0.5. The model was analyzed for the precision and recall in relation to the YOLOv5’s confidence threshold. Figure 1a displays the precision-confidence curve, which demonstrated how the precision increases as the model becomes more confident for its prediction. Additionally, Fig. 1b shows the recall-confidence curve, illustrating that increasing the confidence threshold reduces the likelihood of detecting all objects in an image. The precision and recall were calculated as follows:

1 $$Precision=\frac{TP}{TP+FP},$$

2 $$Recall=\frac{TP}{TP+FN},$$

where *TP* represents the true positives, *FP* represents the false positives, and *FN* represents the false negatives. In this calculation true positives were considered as objects correctly identified within the image, while false positives were considered as incorrect predictions of an object and false negatives were objects missed in comparison to the ground truth annotations.

### RGS classification

**Figure 2:**
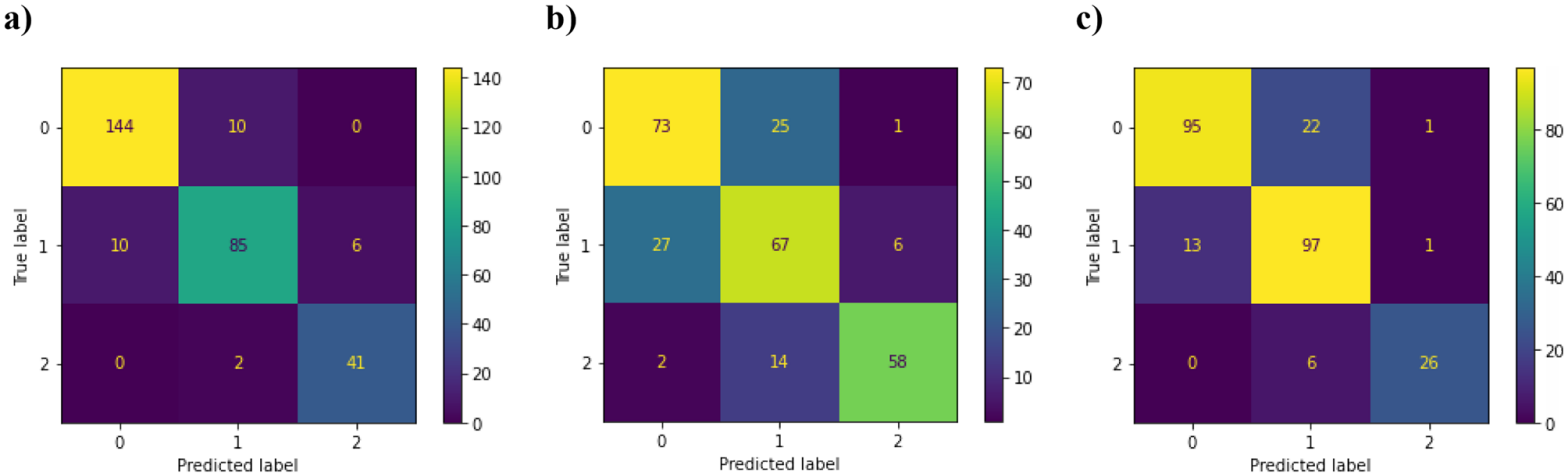
Confusion matrices of ViT based models. (**a**) ViT eye classifier detecting 270 images correctly. (**b**) ViT ear classifier detecting 198 images correctly. (**c**) ViT nose classifier detecting 218 images correctly.

Three vision transformers (ViT) were fine-tuned for the 0, 1, 2 action unit classification. The weighted accuracy, precision, and recall of the three model are reported in Table 1. Weighted accuracy measured the overall accuracy of a model by considering the correctly classified instances in each class as well as the total number of instances of each class in the dataset. This metric is calculated by:

3 $$Weighted \,\,Accuracy=\frac{{N}_{1}{\left[\frac{TP+TN}{TP+TN+FN+FP}\right]}_{1}+ ... {N}_{n}{\left[\frac{TP+TN}{TP+TN+FN+FP}\right]}_{n}}{{N}_{1}+ ... {N}_{n}},$$

where *TN* represents the true negatives and *N* represents the number of instances of the class in the dataset. The results showed that the eye model had the highest overall metrics, while the ear model reported the lowest overall metrics. Despite the differing levels of success, all three models independently reported a weighted accuracy above 0.8. Furthermore, Fig. 2 illustrates the confusion matrices for each ViT, where all models demonstrated success in identifying differences in the appearance of an action unit classified as a 2 compared to an action unit classified as a 0. However, the models performed worse at differentiating the difference between a moderate or uncertain appearance of an action unit which is classified as a 1 compared to other classes.

**Table 1:** Performance metrics for action unit classification models.

Feature	Weighted accuracy	Precision	Recall
Eye	0.930	0.910	0.908
Ear	0.808	0.733	0.725
Nose	0.865	0.844	0.836

**Figure 3:**
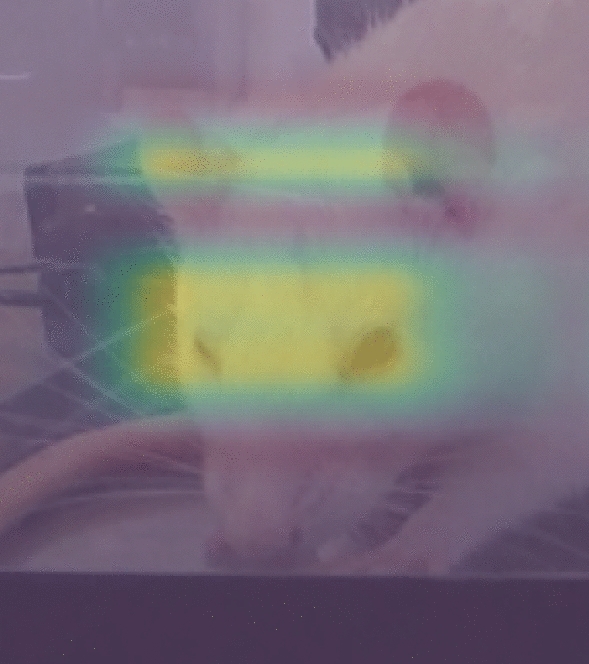
ViT attention heatmap for binary classification.

In addition to training the three action unit classifiers, a fourth ViT was fine-tuned for the binary classification of pain versus no pain. To achieve this, images with a RGS score less than or equal to 0.33 were considered as representing no pain, while images with an RGS score equal to or greater than 1 were considered as pain. The ViT model achieved a weighted accuracy of 0.97. To further analyze the model’s performance, an attention heatmap was created to determine which areas of the image were focused on by the model when inferring the presence of pain, depicted in Fig. 3. The results showed that the model primarily focused on the area around the eyes and the area between the ears in instances of pain indicating that the ears and eyes were better predictors of binary pain, rather than the nose.

### Model validation

**Figure 4:**
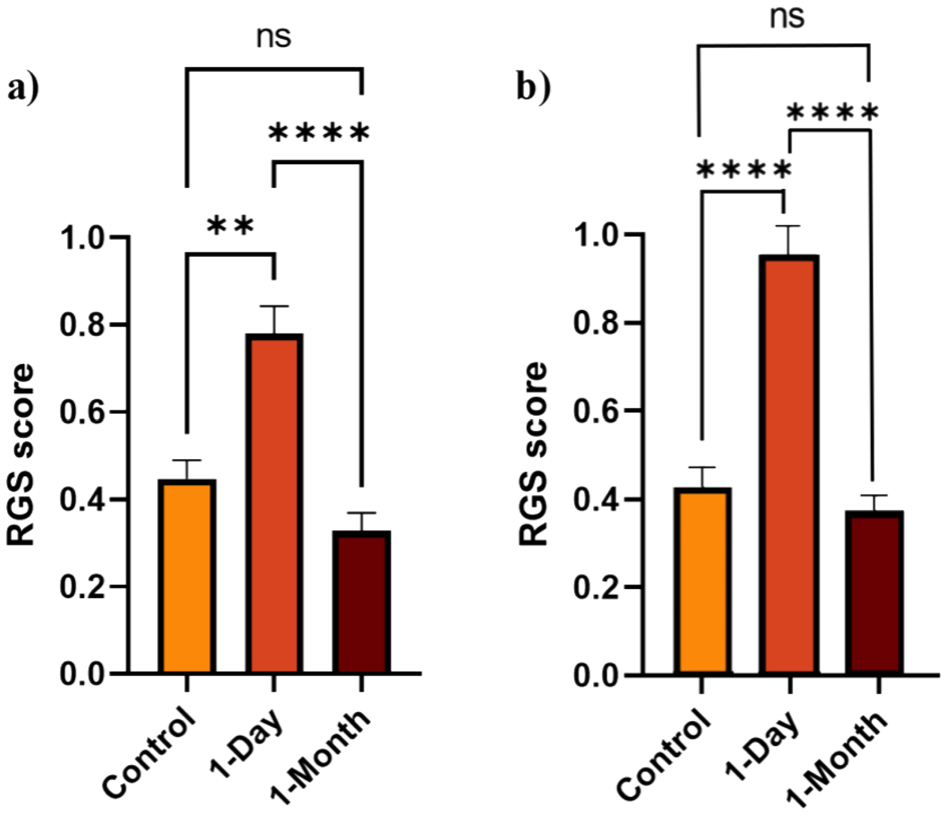
Analysis of mean RGS score of the control, 1-day timepoint, and 1-month timepoint (ns p > 0.05, *p < 0.05, **p < 0.01, ***p < 0.001, ****p < 0.0001): (**a**) Model analysis; (**b**) Experimental analysis. Plotted with SEM bar.

Table 2 provides an overview of the intraclass correlation coefficient (ICC) for each action unit between the four RGS graders for the experimental analysis. The ICC for eyes and final RGS score demonstrated a “very good” reliability between graders, and the ICC for ears and nose both demonstrated a “moderate” reliability^31^. Figure 4 depicts the mean RGS score for the control, 1-day, 1-month images of the bTBI study for both the experimental and aRGS model analysis.

**Table 2:** Intraclass correlation coefficient calculated for each action unit for the validation set between trained RGS graders.

Action unit	ICC
Orbital tightening	0.933
Ear changes	0.568
Nose flattening	0.584
RGS score	0.823

Due to the non-normal distribution of RGS data, the test groups were analyzed non-parametrically. A Kruskal–Wallis test was conducted to evaluate the results of the control images (naïve rats) in comparison to the blast tested rats at 1-day and 1-month images, while a Wilcoxon test was used to compare the repeated measures of the blast group at 1-day versus the 1-month images. Both the experimental RGS analysis (p < 0.0001) and the model analysis (p = 0.001) showed that the dataset from the bTBI study on the 1-day injury animals had significantly elevated RGS scores compared to the control images. Additionally, the RGS score notably dropped in the experimental analysis (p < 0.0001) and model analysis (p < 0.0001) at the 1-month timepoint. The automated model and experimental analysis found that the 1-month image dataset had a lower mean RGS score than the initial control group. However, no significant difference between the two groups was found between the experimental analysis (p = 0.591) and the model analysis (p = 0.224).

**Figure 5:**
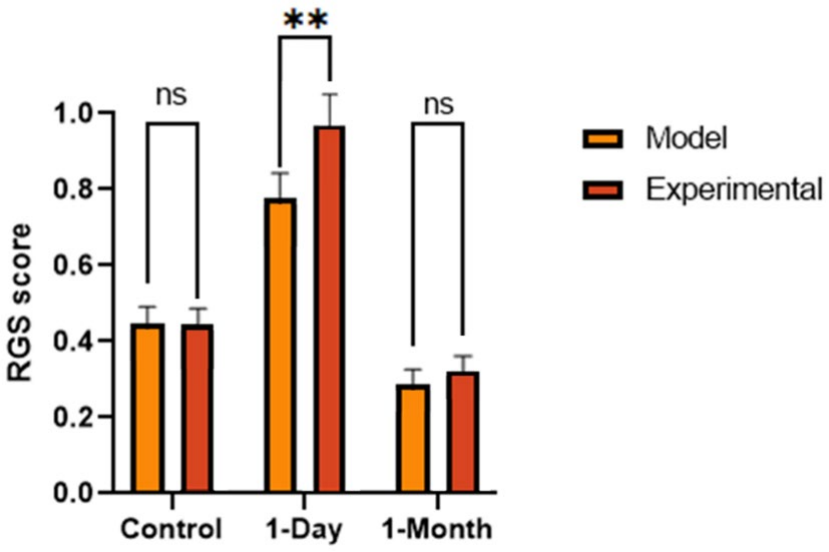
Analysis of the model and experimental RGS results (ns p > 0.05, *p < 0.05). Plotted with SEM bar.

A Wilcoxon test was employed to compare the RGS scores obtained from automated model and manual analysis across all three image groups. The results shown in Fig. 5 display no significant difference between the RGS scores obtained from both methods for the control (p = 0.95) and 1-month (p = 0.270) images, however a significance did appear in the 1-day images (p = 0.006). Furthermore, Table 3 reports the ICC which was calculated in comparison to the experimental analysis for each action unit and total RGS score. Orbital tightening was found to have an ICC greater than 0.9 which indicates a “very good” reliability, while ear changes and nose flattening reported values between 0.61 and 0.80 which indicates “good” reliability. The final RGS score calculated by the model was found to have an ICC of 0.801 when compared to the human graders which would be considered “very good” reliability.

**Table 3:** Intraclass correlation coefficient calculated for each action unit for the validation set results between experimental graders and model predictions.

Action unit	ICC
Orbital tightening	0.935
Ear changes	0.641
Nose flattening	0.624
RGS score	0.801

## Discussion

The aRGS system was developed and provided both an efficient and accurate method for RGS image analysis. The system’s action unit detection achieved a validation accuracy of 97.5%, allowing for 117 of the 120 validation images to be graded for RGS score. Furthermore, the action unit classifiers all produced weighted accuracies above 80%. The holistic RGS model performed similarly to the manual RGS grading but completed the task in 1/14th (3.2 min) of the time required by human graders. In addition, the model successfully identified statistical differences in RGS scores between a control and a bTBI group, as well as recognizing the reduction in pain behaviors 1-month post injury.

While there are no other automated RGS models published, orbital tightening is used in various animal grimace scales^9–13^. Several studies have reported the weighted accuracy of automated classification for orbital tightening in mice, which found values ranging from 0.632 to 0.850^19,25,32^. All of the current methods of automated orbital tightening grading use convolutional neural networks whereas our model utilizes a ViT. The DeepMGS system developed in Chiang et al. performs better than the other convolutional neural networks with an accuracy of 0.85^25^. Despite DeepMGS’s improvements over the other models, the ViT approach outperformed their accuracy by eight percent.

Although orbital tightening is easy to compare to published models, ear changes and nose flattening are difficult to judge without rat specific models. While the MGS includes ears and nose as an action unit area, it primarily focuses on the positioning of the ears and the bulging of the cheek and nose rather than the ear shape and nose flattening. Despite the different interpretations of these facial features, DeepMGS reports an ear position accuracy of 0.780 and nose/cheek bulge accuracy of 0.81, while our model produced an ear change accuracy of 0.808 and nose flattening accuracy of 0.865^25^. Overall, our data indicated that the ViT approach in the aRGS system demonstrated a higher accuracy in comparison to existing deep learning methods. However, additional work using the ViT approach on differing datasets is needed to further compare methods.

The aRGS system not only exhibited higher accuracies than existing machine learning approaches, but it also demonstrated similar inter-rater reliability measured by ICC to human graders^13,19,20^. Across studies that reported interrater reliability with an ICC score, orbital tightening was found to be the most consistent between graders with range in ICC from 0.84 to 0.97. Additionally, ear changes and nose flattening were found to have a “good” to “very good” reliability with a range of 0.62–0.82 and 0.62–0.86 respectively. In the aRGS analysis, the ICC scores were found to fall within the ranges produced by other studies^13,16,19,20^.

While the aRGS model presents a promising solution for efficient and accurate RGS image grading, it has limitations that should be addressed. Firstly, even though the model displayed a strong performance for orbital tightening, ear changes, and nose flattening, it does not consider whiskers. Whiskers were part of the original RGS grading criteria, however, without good lighting and high image quality the whisker becomes very difficult to grade. The GoPro HERO8 black used to film the validation set as well as the Von Frey test was capable of producing image quality that could be used in Rodent Face Finder, however it often left the whiskers blurry. Although whiskers have been omitted in many RGS studies, the exclusion of whisker changes within the model will limit its ability to fully implement the RGS. However, whether the inclusion of whiskers as an action unit in the aRGS would improve the results is still to be determined.

**Figure 6:**
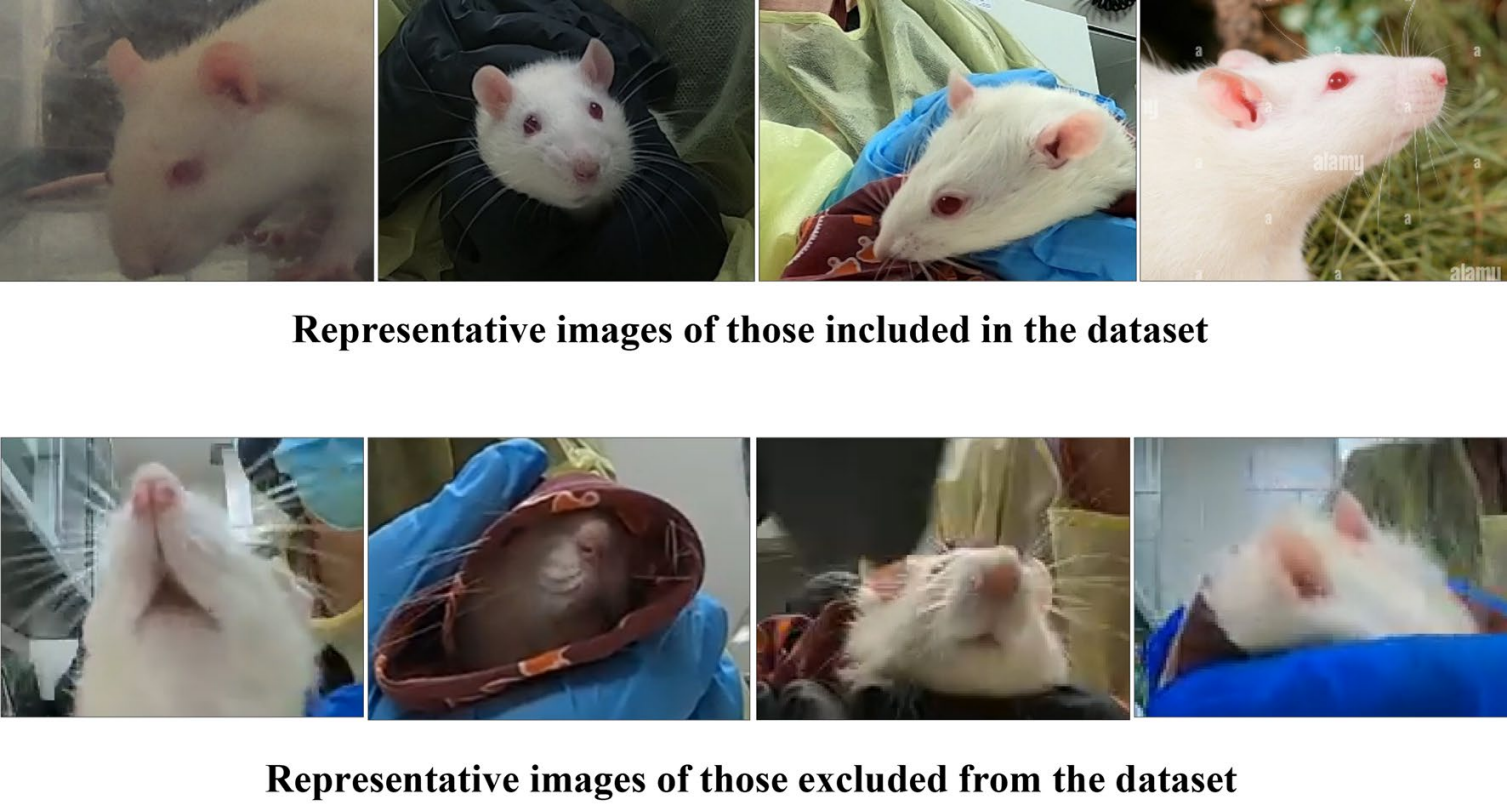
Examples of images included and excluded for the dataset.

Secondly, including more training data would certainly be beneficial. Although the images used in our model were thoughtfully chosen to represent a wide range of scenarios covering different feature categories, backgrounds, lighting conditions, and camera angles as indicated in Fig. 6, we acknowledge that networks will still be susceptible to adversarial perturbations that can mislead any learning system. Thus, it is essential to remain open to further refinement as new data becomes available or the demands of our application grow due to innovation of new methodologies and techniques. With that being said, we hope that by including images with diverse parameters and quality distortions, that this would make our model practical, non-idealized, and applicable in labs where high quality images may not be obtainable.

Furthermore, the ViT action unit classification does not consider the holistic frontal image when grading each action unit. While researchers are advised to consider each action unit separately, they still see the whole context of the image, which may influence the action unit score. The aRGS model is limited to grading the action unit area around the eyes, ears, and nose, therefore it cannot account for the complete image context.

Despite the limitations the aRGS system faces, it provides a promising step towards automated pain behavior monitoring. There are many potential avenues for future research and improvement to the current model, with the aim to eventually develop a real-time, comprehensive, and robust rat welfare monitor. The most straightforward approach is the development of a more robust RGS image database. By improving the current database, the model would become more accurate and generalizable to potential RGS images.

Additionally, an improvement to the current Rodent Face Finder should be made. Currently the Rodent Face Finder application uses Haar cascades to detect one eye and one ear as a means to collect frontal-images with minimum blur. While this method suffices for most images, it does collect images where the nose is not in plain view and requires the video input to run in slow motion to detect objects. One solution for these problems would be implementing the YOLOv5 action unit detector as a frontal image collection system. This system would be capable of detecting all three action units and would have an adjustable confidence threshold. If researchers want to collect clear images, they would be able to set a high confidence threshold and guarantee all three action units are in full view. Furthermore, this method would prevent false negative object detection within the aRGS system, allowing for a more robust grading system.

Although the system was noticeably faster than human graders, it was still unable to grade images in real-time. The model’s average grading speed per image for the validation set was 1.58 s. This was due to the image initially running through the YOLOv5 model, then subsequently running the three to five extracted action unit images through their respective ViT model. When considering each model separately they were all capable of running analysis at real-time, but when combined together are substantially slower. One potential solution for this problem would be to eliminate the ViT models and use the YOLOv5 model as both an object detector and RGS classifier. This strategy may be quick enough to run the program on live video and would consider the holistic image while grading each action, however, it is unknown how effective the model classification would be.

Another solution to developing a real-time automated RGS system would be to use a singular ViT classifier rather than an object detector and action unit specific classifiers. This approach would allow the model to consider the entire context of an image and produce a singular RGS grade. Furthermore, this strategy was attempted as a binary classifier for the RGS and achieved an accuracy of 0.97 while being capable of running on live video. Moreover, the model results were analyzed using an attention heatmap illustrated in Fig. 3, where the model was seen using the action unit areas of orbital tightening and ear changes to classify pain images. This may suggest the model is considering the tightening of the eyes and the distance between the ears when considering a pain classification. Even though this model is able to successfully classify pain images and run noticeably quicker, it is still unable to provide granulated action unit scores. Additionally, the model is susceptible changes in background which would affect the generalizability between research groups.

Nonetheless, the ViT image classifier and attention heatmap may provide interesting research avenues for pain monitoring outside of the RGS. Currently, the RGS considers all four action units equally when making a pain grade. While this works for researchers by making an easily quantifiable measure for pain behavior, it may not be representative of a genuine pain score. For instance, multiple studies have found that orbital tightening is the most robust measure when determining a grimace scale^13,19^. One study that could be conducted to create a more robust pain score is the replication of the pain assay procedure conducted in Langford et al.^9^. However, rather than manually selecting action units, the images could be separated into a control and pain group and classified in a ViT model. After creating the classification model, a post hoc study would be able to use the attention heatmaps and determine what parts of the image are considered when making pain and no pain classifications. This method would not only enable the detection of unrecognized action units in the grimace, but also aid in identifying which current action units are crucial for pain behavior identification and their relative importance.

The development of an automated RGS system for action unit detection and pain behavior classification presents a valuable advancement in the utilization of RGS testing. The system was found to be proficient at correctly scoring RGS images in a fraction of the time when compared to human graders. In addition, the model successfully distinguished differences in action units between a control, day of bTBI rats, and rats with 1-month of recovery suggesting the presence of distinct pain-like behaviors in each group. The findings of this study not only contribute to the standardization of the RGS scores between labs, but also provide a foundation for future research to create a real-time pain monitor and discover new techniques for quantifying pain behaviors. Furthermore, this study presents a promising avenue for pain monitoring beyond the RGS in other animal models. In conclusion, this research represents a significant step towards overcoming the barriers for the utilization of RGS testing and demonstrating the feasibility of real-time grimace monitoring.

## Methods

All animal experiment protocols described within this study were approved by the Virginia Tech Institutional Animal Care and Use Committee and conducted in accordance with the ARRIVE guidelines for reporting experiments involving animals^33^. All experiments were performed within the applicable regulations and guidelines established.

### Model development

#### Feature detection

Action unit feature detection was performed by fine-tuning a YOLOv5 small (YOLOv5s) model from Ultralytics^34^. The model used a Leaky ReLU activation function within the hidden layers, and a sigmoid activation function for the final detection. Additionally, the model used stochastic gradient descent (SGD) with momentum for the optimization function.

YOLOv5 calculated three loss functions including (1) box regression loss to determine how well the predicted bounding box covers the ground truth box (2) objectness loss to measure the probability an object is within the proposed region and (3) class loss for how well the algorithm is predicting the correct object class. The objectness and class loss both used a binary cross-entropy loss function where if no ground truth exists for a predicted bounding box only objectness loss was affected. The box regression loss was calculated using generalized union of intersection (GIoU). The overall loss function used in YOLOv5 was the weighted sum between the three losses, where box loss was weighted as 0.05, object loss was weighted as 1, and class loss was weighted as 0.5.

The YOLOv5s model was pre-trained using the COCO database, it was then fine-tuned for action unit detection using the 1122 frontal-facing rat images and their respective annotations. The model was trained for 100 epochs using mosaic augmentation to provide samples with a diverse set of objects within the image that vary in size and location. To combat overfitting, the epoch with the lowest total validation loss was taken as the final model for the action unit object detector.

#### Pain classification

Individual classification models were developed for action unit specific pain grading, where each action unit model was built to predict pain levels on a standard RGS scale of 0–2. The action unit classifiers were constructed using an open-source transformers package from hugging face^35^. The open-source ViT model utilized the original ViT architecture discussed in Dosovitskiy et al.^36^. In these models, the images are preprocessed to a size of 224 × 224 pixels and normalized. The ViT models employed a 16 × 16 patch size for a total of 196 patches and runs through 12 transformer encoders composed of self-attention and feed forward layers. The ViT used SGD optimization and a weighted cross-entropy loss due to a large class imbalance between the action unit RGS scores. Additionally, the ViT uses gaussian error linear units (GELU) and SoftMax activation functions.

The ViT models underwent initial pre-training utilizing the ImageNet-21 k database, followed by subsequent fine-tuning using the established action unit databases for the eye, ear, or nose. The training process was run for a duration of 20 epochs, where the model state with the best validation loss was selected as the final action unit classifier for the aRGS system. Moreover, a ViT model was fine-tuned with the original RGS images using a binary pain vs. no pain scale where RGS below ≤ 0.33 was no pain and ≥ 1 was classified as pain.

### Animals/blast exposure

Male 10-week-old Sprague Dawley rats (Envigo; Dublin, VA) were used (n = 10). Prior to blast testing, animals weighed between 250 and 300 g and were acclimated for seven days (12 h dark/light cycle) with food and water provided ad libitum*.*

Blast waves were generated using a custom Advanced Blast Simulator (ABS) (Stumptown; Black Mountain, NC) located in the Center for Injury Biomechanics at Virginia Tech. The ABS system consists of three sections that produce and dissipate a blast wave. The blast wave was created through a helium-driven rupture of a membrane that was then dissipated in an end-wave eliminator. The result was a single peak overpressure to represent a free-field blast wave. The pressure measurements were collected using a Dash 8HF data acquisition system (AstroNova TMX; West Warwick, RI).

Rats were anesthetized with isoflurane and were placed in a mesh sling within the ABS system, in the prone position facing the blast wave. Each animal was exposed to three static overpressure insults (19.048 psi ± 0.272) separated by 1 h (3 × 1 h).

### Rat grimace scale (RGS)

The RGS study utilized two experimental groups consisting of uninjured control rats (n = 4) and injured rats (n = 4) from the blast exposure protocol. Rats were individually recorded with a single GoPro HERO8 Black at the long end of a standard transparent cage. The three walls without the camera were covered to be opaque to entice the rat to face towards the camera. Each rat was recorded for 15 min, and frontal-facing images were captured using Rodent Face Finder® from Sotocinal et al.^13^. One image was taken every 1.5 min for a total of 10 images per rat. Additionally, the injured rats underwent RGS analysis at two timepoints of at the day of injury, and 1-month post injury for a total of 120 images.

The images from the validation experiment were randomized by a third-party blinded to the original test groups and placed on separate PowerPoint slides. The images were then graded for RGS by four trained researchers with various scientific backgrounds for orbital tightening, ear changes, and nose flattening. After manually grading the images, the 120 samples were run through the aRGS system and recorded for total computation time, total RGS score, as well as the granulated action unit scores.

### Statistical analysis

Statistical analysis was conducted using GraphPad Prism 9 software. Due to the non-normal distribution of RGS data, the test groups were analyzed non-parametrically. A Wilcoxon test was used to compare the experimentally graded images to the model images. Furthermore, a Kruskal–Wallis test was used to compare the control images, to the 1-day and 1-month post-injury images, while an additional Wilcoxon test was used to compare the 1-day and 1-month groups. Differences were considered statistically significant if the p-value < 0.05.

Additionally, the intraclass correlation coefficient (ICC) was calculated between the model and the experimental validation grades using a one-way random effect, absolute agreement model. Moreover, ICC was calculated for the four graders to compare intergrader reliability. The interpretation of the ICC was based on Landis & Koch (1977), where a score > 0.81 was considered “very good”, 0.8–0.61 was “good”, 0.6–0.41 was “moderate”, 0.4–0.21 was “fair”, and anything < 0.2 was “poor”^31^.

## Data Availability

The datasets generated and/or analyzed from the current study are available upon request. The aRGS model generated during the current study is available in the GitHub repository, https://github.com/Barn99/Automated-RGS.
